# Editorial: Pharmacological Approaches Targeting Neutrophilic Inflammation

**DOI:** 10.3389/fphar.2021.763140

**Published:** 2021-09-13

**Authors:** Boris V. Chernyak, Alexey V. Sokolov, Tsong-Long Hwang, Roman A. Zinovkin, Galina F. Sud’ina

**Affiliations:** ^1^ Belozersky Institute of Physico-Chemical Biology, Lomonosov Moscow State University, Moscow, Russia; ^2^ Institute of Experimental Medicine, St. Petersburg, Russia; ^3^ Graduate Institute of Natural Products, Chang Gung University, Taoyuan, Taiwan; ^4^ Graduate Institute of Health Industry Technology, Chang Gung University of Science and Technology, Taoyuan, Taiwan; ^5^ Department of Anesthesiology, Chang Gung Memorial Hospital, Taoyuan, Taiwan

**Keywords:** neutrophils, inflammation, cancer-related inflammation, neutrophil-derived molecules, neutrophil extracellular traps (NETs)

Neutrophils are actively involved in the protection of our body against pathogen infection, but the consequences of their activation contribute significantly to pathogenesis of various diseases. This is why neutrophil-mediated responses should be finely tuned, and therapeutic interventions should balance between targeting neutrophil functions and uncontrolled neutrophil activation. Several decades ago, Elmer Becker wrote a great article: “The short and happy life of neutrophil activation,” where he described the importance of neutrophil functions to human physiology (Becker 1990). The novel coronavirus pandemic COVID-19 again demonstrated a critical role of neutrophils. An increased neutrophil-to-lymphocyte ratio (Liu et al., 2020) and the release of neutrophil extracellular traps (NETs) (Zuo et al., 2020) predicted severe illness at SARS-CoV-2 infection. Excessive NETs formation can be involved in the development of the «cytokine storm» and immunothrombosis, which are the main cause of severe complications associated with COVID-19. Therefore, neutrophil can be a pathogenic marker and drug target of COVID-19 (for review see Chiang et al. (2020)). A detailed study of these cells is extremely important for medicine and pharmacology, and the aim of this topic is to gather new trends in pharmacological approaches targeting neutrophilic inflammation.

Neutrophils perform their functions through various mechanisms, including chemotaxis, phagocytosis, production of reactive oxygen species (ROS), and physiologically active molecules. Recently it was shown that mitochondrial ROS play a key role in neutrophil activation including NETs formation (Vorobjeva et al., 2017; Vorobjeva et al., 2020). In the opinion article presented by Vorobjeva et al., the authors suggest that targeting mitochondrial ROS may be a beneficial approach in the treatment of neutrophilic inflammation in severe pneumonia including COVID-19.

Neutrophils are key players in a dysregulated host response to sepsis infection. Neutrophil migration and antimicrobial activity are impaired while NET release is increased. This results in an inappropriate response to primary infection and potentially increases the susceptibility to secondary infection. In sepsis, immature neutrophils are accumulated in peripheral blood and serve as a biomarker predicting serious bacterial infection (for review see Denning et al. (2019)). Molecules that target neutrophil maturation and function are an important research field for the evaluation of perspective sepsis treatment. The impact of different neutrophil subsets in pathogenesis of sepsis and possible pharmacological approaches selectively targeting neutrophils that mainly mediate the tissue injury are discussed in the paper of Shen et al. from this research topic.

Neutrophil heterogeneity and therapeutic strategies based on neutrophil heterogeneity attract attention of many investigators. The phenotypic diversity of neutrophils contributes to their immune responses, the production of NETs, and their own clearance. In addition to the classification based on surface molecules, neutrophils also differ in their buoyant density. Low-density granulocytes is a discrete population that may manifest either immunosuppressive or proinflammatory properties (Ley et al., 2018). In autoimmune diseases, these neutrophils spontaneously release NETs leading to vascular injury and exacerbated inflammation. Therapeutic strategies based on this pathogenesis are discussed by Nakazawa and Kudo in their opinion paper.

Pharmacological modulations of neutrophil functions are reviewed in this collection by Gierlikowska et al. The action of plant extracts, plant-derived compounds, and synthetic drugs on phagocytosis, degranulation, and formation of NETs by neutrophils is discussed in detail. Influence of plant-derived compounds on immunomodulatory neutrophil functions is a hot topic. In the original research of Korinek et al., the volatile oils from fennel and cumin were screened for various biological activities and it was found that these essential oils significantly suppressed the activation of human neutrophils.

As an original contribution to this field, Felix et al. presented in this collection an interesting report about using natural flavonoid compound biochanin A (BCA) in the resolution of inflammation in arthritis, where neutrophils are the main inflammatory cells. The authors observed for the first time that BCA promotes resolution of inflammation by decreasing neutrophil accumulation, enhancing neutrophil apoptosis, and stimulating efferocytosis.

Neutrophil rapid recruitment into inflamed tissues, the elimination of invading microorganisms, and tissue repair are critical for host defense. All these processes are mediated by neutrophil adhesive interactions. The integrin receptors and neutrophil adhesion are the main regulators in the shaping innate and adaptive immune responses (for review see Margraf et al. (2019)). Penetration of neutrophils into the central nervous system, their adhesion, and concomitant secretion can contribute to inflammatory reactions and pathogenesis of many neurological and neurodegenerative diseases (Feng et al., 2020; Passaro et al., 2021). In the current topic, Galkina et al. have studied the inhibitory effect of antidepressant imipramine on the adhesion and accompanied secretion of neutrophils in the model of integrin-dependent neutrophil infiltration. This effect may contribute to the antidepressant action of the imipramine and indicates its anti-inflammatory potential in the treatment of neurological diseases.

Neutrophils recruited into cancer tissue are immunosuppressive and contribute to cancer progression (for review see Jaillon et al. (2020)). Anderson et al. in this research topic discussed movement of neutrophils into the tumor microenvironment and their role in the pathogenesis of non-small cell lung cancer (NSCLC). Recent studies, both pre-clinical and clinical, demonstrated that neutrophils in NSCLC microenvironment not only stimulate tumor growth and spread, but also significantly interfere with anti-tumor therapy and especially novel immunotherapies. Described mechanisms of tumor-associated neutrophil recruitment, their heterogeneity and activity demonstrate the high potential of neutrophil-targeted pharmacological strategies as an addition to standard anti-cancer therapies.

**SCHEME 1:**
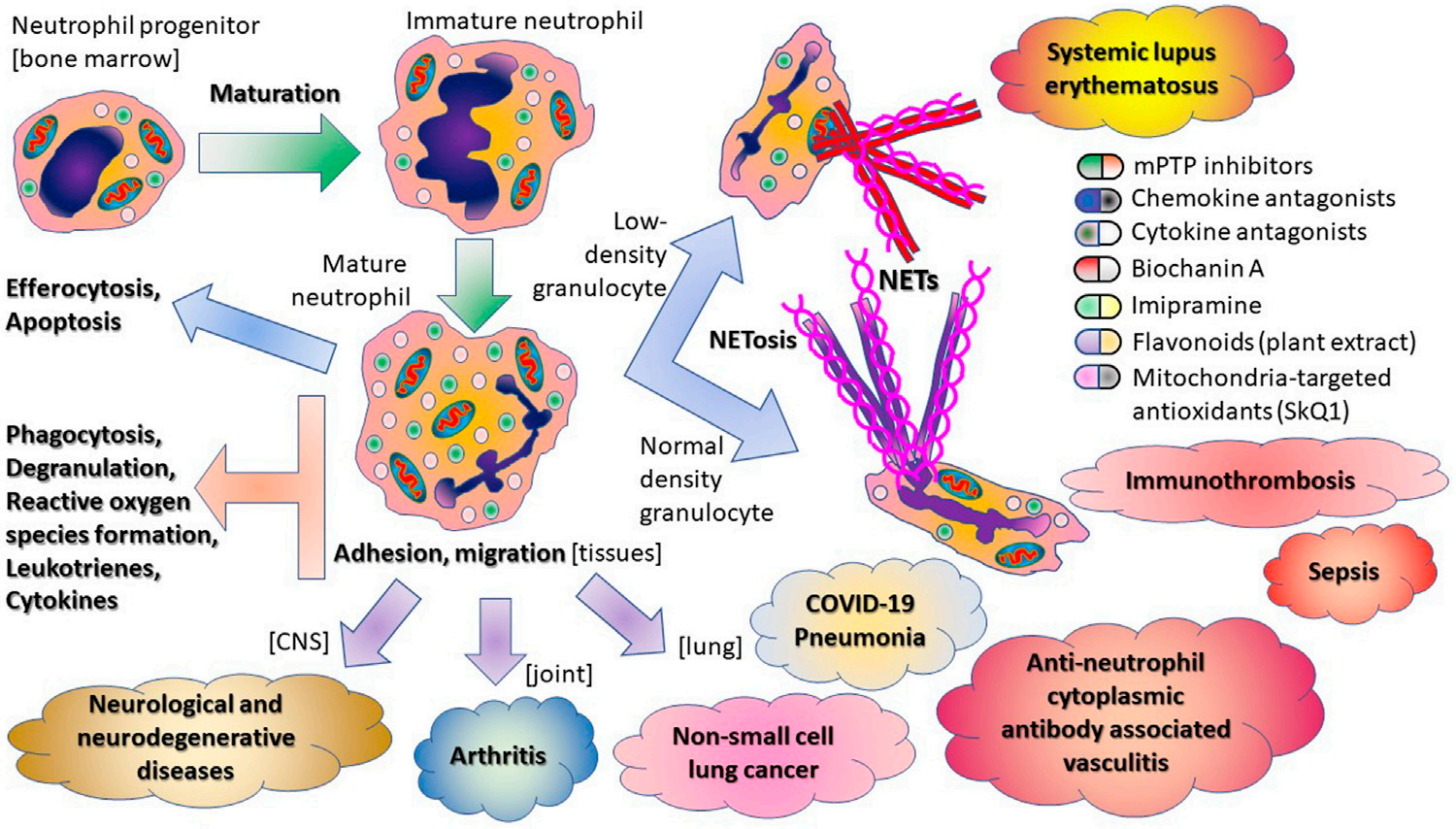
Multifaceted activity of neutrophils in host immunity.

In summary, the papers in this topic issue illustrate the multifaceted activity of neutrophils in host immunity (Scheme 1).
